# Extension of the NIST AC-DC Difference Calibration Service for Current to 100 kHz

**DOI:** 10.6028/jres.102.007

**Published:** 1997

**Authors:** Joseph R. Kinard, Thomas E. Lipe, Clifton B. Childers

**Affiliations:** National Institute of Standards and Technology, Gaithersburg, MD 20899-0001

**Keywords:** ac current, ac-dc difference, current calibrations, current shunts, thermal converter, thermal current converter, thermoelement

## Abstract

The NIST calibration service for ac-dc difference of thermal current converters relies on multijunction thermal converters as the primary standards, and various thermal converters and thermoelements (TEs) as the reference and working standards. Calibrations are performed by comparing the ac-dc difference of a customer’s thermal current converter to the ac-dc difference of a NIST standard current converter. Typical artifacts accepted for calibration include single-junction thermoelements, multijunction thermal converters, and transfer shunts for use with TEs. This paper describes the standards on which the calibration service is based and the results of the study to characterize the NIST standards over the extended frequency range from 50 kHz to 100 kHz at currents from 1 mA to 20 A. The general method for the frequency extension at high frequency involves the use of thermoelements in the 5 mA range, with small frequency dependence, as the starting point for build-up and build-down chains to cover the whole range from 1 mA to 20 A.

## 1. Primary Standards

The NIST primary standards of ac-dc difference for current are multijunction thermal converters (MJTCs). The primary MJTC group consists of six converters with rated heater currents ranging from 5 mA to 50 mA. The ac-dc differences of these MJTCs are believed to be below 0.5 μA/A at frequencies ranging from 30 Hz to 10 kHz. Detailed descriptions of the primary MJTCs and the results of their intercomparisons have been published previously [1,2].

## 2. Extension to 100 kHz

The existing characterization of thermal current converters (TCCs) for frequencies above the range of the primary MJTCs, i.e., from 10 kHz to 50 kHz, relies on the frequency flatness of the ac-dc difference for selected, specially constructed, single-junction thermoelements. The single-junction thermoelements (TEs) which were chosen for use in extending the frequency up to 50 kHz, and the methods of characterization, have been described before [1,3]. Single-junction TEs were also chosen for this project to extend the ac-dc difference characterizations up to 100 kHz because they have a much simpler structure than the MJTCs and can be constructed to have smaller reactances and therefore wider frequency ranges.

Four 5 mA TEs, identified as FX_2_, FY_2_, FA, and FB, have been compared as current converters up to 100 kHz by one of two methods. In the first method, the heaters of the two TEs are connected in series using interconnection wiring intended to minimize any leakage current. This arrangement may suffer from bead-effect error, as described by Hermach [1], so a second method was also used. In this method, the two TEs were assembled in coaxial structures with two nearly identical 5.6 kΩ resistors in series with the heaters to form thermal voltage converters and the resistor-TE combinations compared as such. In this arrangement, the TEs are working as current converters, so ac-dc differences for current are obtained. The series resistors were chosen to have very small differences in reactance; by interchanging them and taking the average, the residual reactance errors are nearly eliminated.

## 3. Extension of Current Ranges

The characterization of standard TCCs at higher and lower currents is based on a process of build-up and build-down through the current levels. These methods have been described previously [1,3,4] and will only be summarized in this paper. Range-to-range intercomparisons have been made by techniques employing interconnection arrangements essentially the same as those described above for the frequency extension [1,3,6]. For lower current ranges, two TEs were compared in series and then connected in parallel using two nearly identical resistors to make the currents sufficiently matched in phase [1]. For this method, the TEs are used at or near their rated current so current-level dependence is essentially eliminated from the characterization process. At higher currents, generally greater than a few tens of milliamperes, TEs of different ranges were compared in series at current levels suitable for the lower range TE. Level dependence was tested by making comparisons at multiple levels and by cross checks between different build-up paths.

## 4. NIST Reference and Working Standards

Although NIST does use some current shunt-TE combinations as check standards and transfer standards, essentially all reference and working standards are thermoelements. As standards, TEs are inherently superior to shunts because they have smaller structural reactances and stray impedances and may be less affected by thermal drift. These characteristics yield ac-dc differences which are generally flatter with frequency and less dependent on surrounding structures and operating current. Furthermore, in any structure where currents divide, such as a shunt in parallel with a TE, it is never possible to make the ac current division identical to the dc current division at all frequencies since the ac divides according to impedance ratios and the dc according to resistance ratios.

**Fig. 1:**
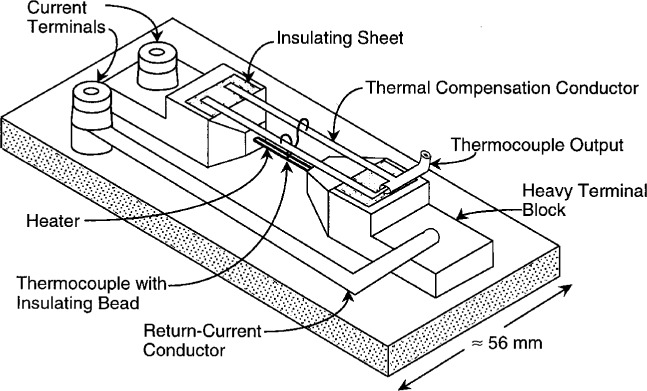
Diagram of a typical high-current TE for currents from 500 mA to 20 A. These TEs are specially constructed with low-inductance, return-current conductors; thermally-lagged, Evanohm heaters; and thermal compensation.

For currents at 250 mA and below, the standards are single-junction, vacuum thermoelements. Many of these TEs are specially constructed with Evanohm^1^ heaters. For currents from 500 mA to 20 A, special air-mounted thermoelements with thermally lagged heaters and thermal compensation are used [8]. A diagram of a typical high-current TE is shown in Fig. 1. For the highest currents, tubular heater structures are used because of skin effect. Electrically insulated, thermal compensation straps between the thermocouple output leads and the heavy terminal blocks on the ends of the heater significantly reduce the variation in output emf due to the thermal drift and warm-up of the heater and ambient temperature variations. A summary of the approximate characteristics for the TEs in both the reference and working sets is given in Tables 1a and 1b.

For this work, one transfer shunt was used in the build-up path at the 6 A level. This 6 A shunt was of a special design and has been studied carefully [3].

## 5. Results

**Fig. 2:**
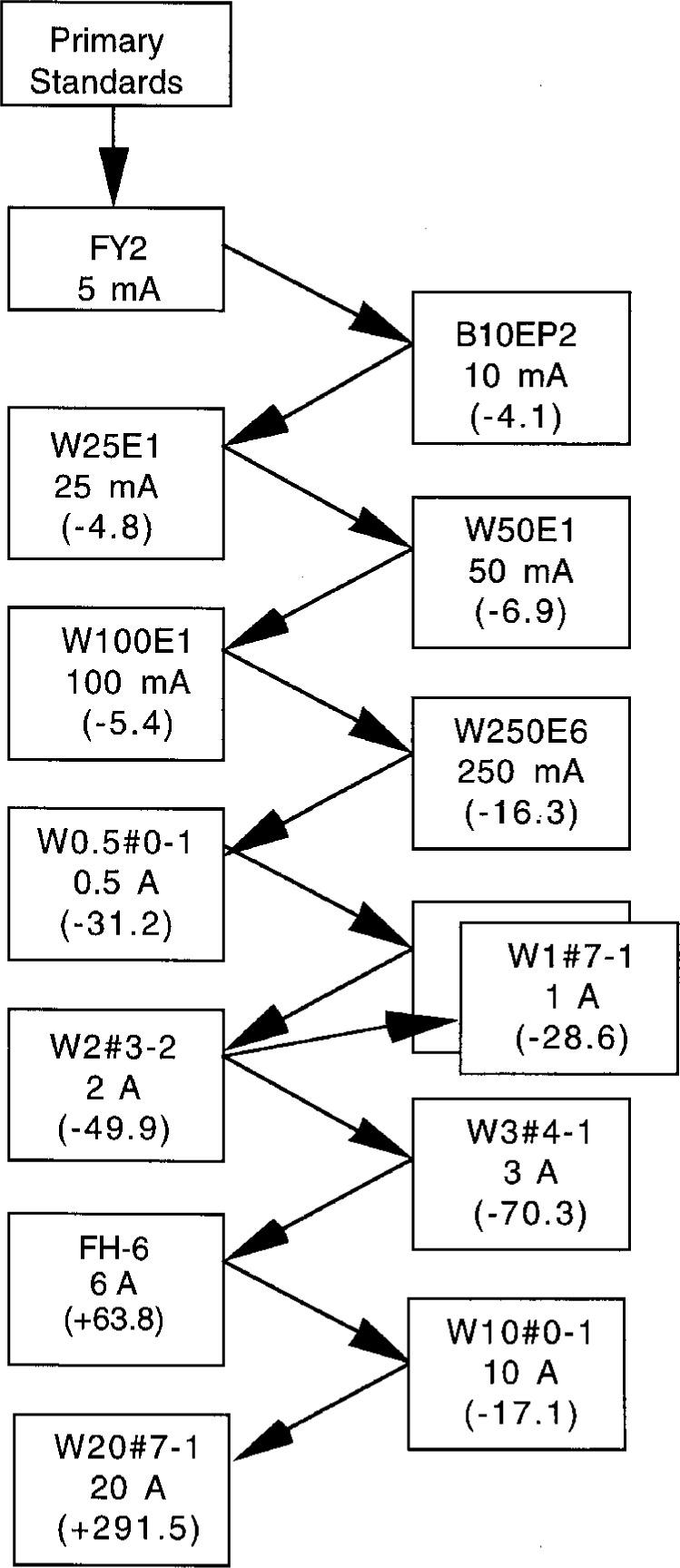
Results of the build-up process for the intercomparison of the reference standards from 5 mA to 20 A at 100 kHz. The arrows point to the converters in the “test” position, and the measured ac-dc differences (shown in parentheses) are given in microamperes per ampere (μA/A). At the 1 A level, the converter that was used to perform the build-up failed shortly after the process and is no longer available. That converter is represented by the blank box covered by converter W1#7-1, the unit presently in the set.

Typical results of the build-up process for the intercomparison of the reference standards from 5 mA to 20 A are given in Fig. 2 for 100 kHz. The arrows point to the converters in the “test” position, and the measured ac-dc differences are given in microamperes-per-ampere (μA/A). Generally the comparisons were made at the rated current for the lower of the two converters. At the 1 A level, the converter that was used to perform the build-up failed shortly after the measurements and is no longer available. That converter is represented by the blank box covered by converter W1#7-1, the unit presently in the set. The ac-dc difference of W1#7-1 varies more with the level of the applied current than the previous converter, so the most recent data taken with the missing converter is being used. Steps are being taken to better characterize the existing units or to replace them with new converters.

**Fig. 3:**
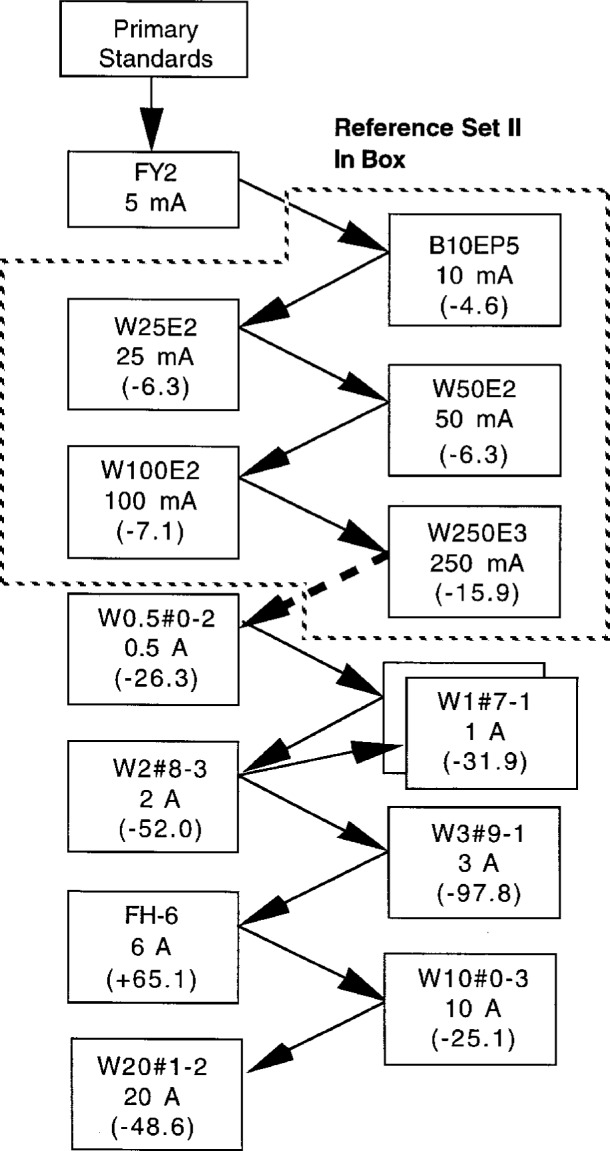
Results for a second build-up process using the working standards from 0.5 A to 20 A combined with a second set of backup reference TEs from 5 mA to 250 mA. The step from 250 mA to 500 mA is shown dashed because the comparison involved, for convenience, the measurement of an intermediate converter. A converter which is presently unavailable was used at the 1 A level, and the unit now in the set and shown in the box is W1#7-1. The ac-dc differences are given in μA/A.

**Fig. 4:**
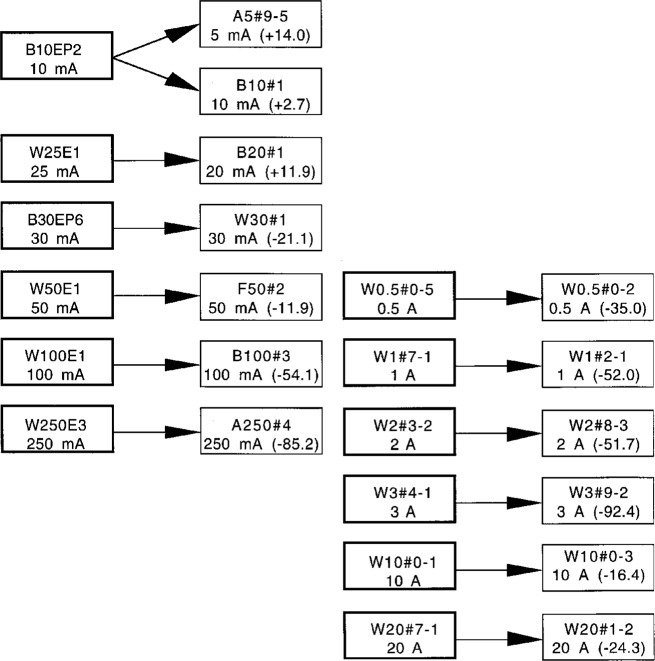
Crossover or direct comparison between the reference standards and the working standards at rated current, in μA/A.

Typical results for a second build-up process using the working standards from 0.5 A to 20 A and a second set of backup reference TEs from 5 mA to 250 mA are shown in Fig. 3. The regular working standards for 250 mA and below are switch selected and are all located in the same box; therefore it is not possible to measure them in a build-up process. To confirm the accuracy of the build-up methods, a direct comparison between the reference standards and the working standards at rated current was performed. Figure 4 presents the ac-dc differences measured for the working standards as a result of this direct comparison. The step from 250 mA to 500 mA is shown as a dashed line in Fig. 3 because this involved, for convenience, the measurement of an intermediate converter. A converter which is presently unavailable was used at the 1 A level, as described in the above paragraph. The unit now in the set is shown in the box as W1#7-1. Converter W1#7-1 is shown with slightly different values in Figs. 2 and 3 owing to the two different build-up paths.

## 6. Uncertainty Analysis

The analysis and combination of uncertainties for this work have been done according to NIST Technical Note 1297 [7]. The uncertainty analysis for the 5 mA TEs that were used as primary standards for the frequency extension up to 100 kHz is described in detail in Ref. [1]. Both the type A uncertainties (those evaluated by statistical means) and the type B uncertainties (those evaluated by other means) are described in Ref. [1]. For these primary thermoelements, the final root-sum-square combination and expanded uncertainty, for a coverage factor of *k* = 2, are given as the primary standard elements in Table 2. Included in this analysis were:

- contributions for the NIST primary standard multijunction thermal converters at frequencies up to 10 kHz;
- contributions for the characterization of the reference standards up to 50 kHz;
- pooled standard deviations for characterization of reference standard thermoelements;
- contributions to the variation of ac-dc difference with frequency to 100 kHz for reference TEs including current definition, skin effect, and bead-effect error.

Table 2 also gives the summary of the uncertainty analysis for the build-up process and the characterization of the group termed “reference standard thermoelements.” The table contains contributions for both type A and B uncertainties including:

- pooled standard deviations for the comparator;
- uncertainties for the comparator system;
- variation of ac-dc difference with current level;
- contributions to the ac-dc difference from dielectric loss in the insulating bead and from RF pickup;
- variation in ac current due to proximity effect;
- reproducibility of circuit and current configuration from one physical assembly to the next.

The uncertainty for each step is calculated as the square root of the sum-of-the-squares (RSS) of these contributions and the *k* = 1 RSS uncertainty of the previous step.

Table 3 gives the summary of the uncertainty analysis for the characterization of working standards by direct comparison to a reference standard, called a crossover measurement, rather than by a build-up process. The table contains contributions for both type A and B uncertainties including:

- pooled standard deviations for the comparator;
- uncertainties for the comparator system;
- stability of the working standard TE;
- contributions to the ac-dc difference from dielectric loss in the insulating bead and from RF pickup;
- variation in ac current due to proximity effect;
- reproducibility of circuit and current configuration from one physical assembly to the next.

The uncertainty for each step is calculated as the square root of the sum-of-the-squares (RSS) of these contributions and the *k* = 1 RSS uncertainty of the previous step.

Table 4 gives the summary of the uncertainty analysis for the measurement of a client’s transfer shunt by direct comparison to a working standard. The table contains contributions for both type A and B uncertainties including:

- pooled standard deviations for the comparator;
- uncertainties for the comparator system including variations in measured data associated with different ac current sources;
- uncertainty of the definition of the shunt’s characteristics;
- contributions to the ac-dc difference from dielectric loss in the insulating bead and from RF pickup;
- variation in ac current due to proximity effect;
- uncertainty due to thermal drift of shunt’s characteristics during measurement.

Table 4 reflects the fact that the behavior of transfer shunts at 100 kHz differs considerably from that of thermoelements. For example, the build-up process for shunts is in general less reliable than for TEs. The performance of shunts is more dependent on the stray impedances to ground. Shunts also display relatively minor, but quite observable, variations in ac-dc difference as a function of the specific ac source used. As might be expected, this dependance on the ac source increases with current level and frequency. The analysis in Table 3 includes allowances for these variations which are the subject of continuing study.

**Fig. 5:**
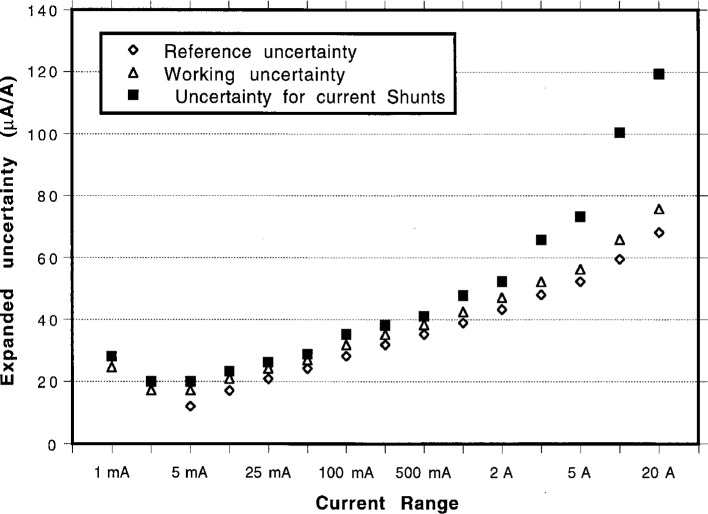
Final, expanded uncertainties (in μA/A) for thermal current converters at 100 kHz, calculated with a coverage factor of *k* = 2, plotted together.

**Fig. 6:**
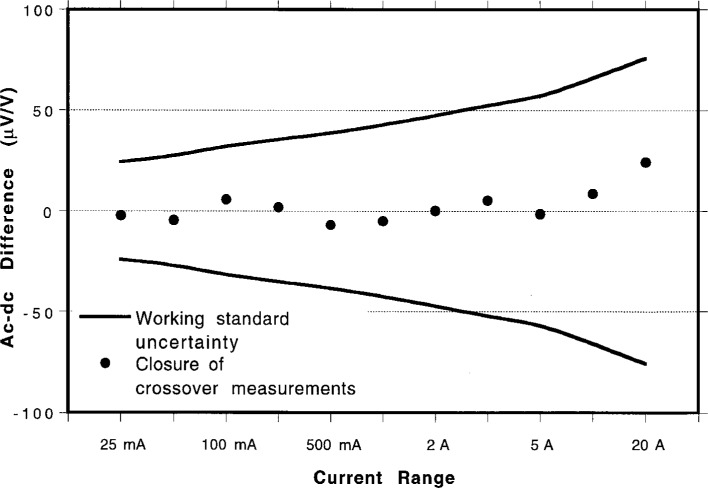
Expanded uncertainties (in μA/A) for the reference standards plotted with the closure between the buildup paths.

The final, expanded uncertainties shown in Tables 2, 3, and 4 were calculated with a coverage factor of *k* = 2 for each of the above analyses and are plotted together in Fig. 5. To show how the variations between the two build-up paths used to characterize the standards compare to the calibration service uncertainties developed in this paper, the quantity *Δ* was calculated from the equation

$$\Delta =\delta_{c}-\left(\delta_{w}-\delta_{r}\right),$$ (1)

where *Δ* represents the closure of the two path, *δ*_c_ is the uncorrected ac-dc difference of the crossover measurement, *δ*_w_ is the ac-dc difference assigned to the working TCC by the working converter build-up path, and *δ*_r_ is the ac-dc difference of the reference TCC assigned by the reference converter build-up path. The expanded uncertainties for the working standard TCCs along with the closure between the two build-up paths are presented in Table 5 and plotted in Fig. 6. For perfect agreement between the two methods, all values of *Δ* would be 0. The discrepancy between the two build-up paths is well below the working standard uncertainty in all cases which is itself smaller than the uncertainty provided in a calibration test report, as given in Table 4.

Determinations of ac-dc difference for thermal current converters and transfer current shunts at 100 kHz require extra attention to good measurement practices. For example, in all such measurements every connection in the current circuit must be screwed down tightly and maintained tight throughout the process; otherwise the results are unstable. On ranges above about 2 A, the delay time between switching and data collection must be increased from about 30 s to 40 s, to around 40 s to 60 s, to allow the sources and measurement system to settle. The increased measurement difficulties and errors at the current and frequency extremes are evident in the escalation of the uncertainties as shown in Fig. 5.

## 7. Conclusion

The NIST thermal current converter standards have been characterized for currents up to 20 A at 100 kHz. The uncertainties for calibrating current converters and transfer shunts have been calculated using the root-sum-square approach in accordance with the requirements of NIST and the Comité International des Poids et Mesures (CIPM). These new uncertainties will be available for routine calibrations in the near future.

## Figures and Tables

**Table 1a:** Characteristics for low-current thermoelements. All heaters are Evanohm wire mounted in vacuum. All thermocouples are chromel-constantan. Bead insulation resistance is >10^10^ Ω for all of the thermoelements

Range (mA)	Heater resistance (Ω)	Thermocouple resistance (Ω)	Output emf (mV)	Reversal error (μA/A)	Time constant (s)
5	50 to 60	8 to 13	11	50	<2
10	30 to 40	7 to 8	9	220	<2
20	20 to 25	3 to 5	9	250	<2
30	6 to 7	3 to 5	9	240	<2
50	3 to 4	3 to 5	9	100	<2
100	1 to 2	3 to 5	10	140	<2
250	0.5 to 1.0	2 to 4	11	130	<3

**Table 1b:** Characteristics for high-current thermoelements. Heaters are Evanohm wire up to 10 A. The heaters for 10 A and 20 A are tubes formed from Evanohm strip. All thermocouples are chromel-constantan. See Fig. 1 for a typical design. Bead insulation resistance is >10^10^ Ω for all of the thermoelements

Range (mA)	Heater resistance (Ω)	Thermocouple resistance (Ω)	Output emf (mV)	Reversal error (μA/A)	Time constant (s)
0.5	0.5	10 to 11	9	30	<2
1.0	0.2	10 to 11	13	75	<2
2.0	0.1	9 to 11	12	80	<2
3.0	0.06	9 to 16	11	150	<2
10.0	0.02	14 to 16	9	150	<2
20.0	0.008	11 to 14	10	80	<2

**Table 2:** Uncertainty analysis (in μA/A) for NIST ac-dc difference reference standards. The uncertainties are given to the nearest 0.1 μA/A for consistency, but this precision may not always be significant or meaningful

	Pooled standard deviation	Comparator and sources	Variation with level	Bead & RFI	Proximity effect	Repro-ducibility	RSS uncertainty	Expanded uncertainty
Primary							0.7	1.4
5 mA	1.5	1.2	1.2	2.9	1.2	4.6	6.1	12.1
10 mA	1.5	1.2	1.2	2.9	1.2	4.6	8.5	17.1
25 mA	1.5	1.2	1.2	2.9	1.2	4.6	10.4	20.9
50 mA	1.5	1.2	1.2	2.9	1.2	4.6	12.0	24.1
100 mA	1.5	1.2	1.2	3.5	1.7	5.8	14.1	28.2
250 mA	2.0	1.2	1.2	3.5	1.7	5.8	16.0	31.9
500 mA	2.0	1.2	1.2	3.5	1.7	5.8	17.6	35.2
1 A	2.0	1.2	1.2	3.5	1.7	6.9	19.5	39.0
2 A	2.0	2.3	2.9	4.0	2.9	6.9	21.7	43.3
3 A	2.0	2.3	2.9	4.0	2.9	8.1	24.0	48.1
6 A	2.0	2.3	2.9	4.0	2.9	8.1	26.2	52.3
10 A	3.0	2.3	4.0	4.6	4.0	11.5	29.7	59.5
20 A	3.0	2.3	4.0	4.6	4.0	14.4	34.1	68.1

**Table 3:** Uncertainty analysis (in μA/A) for NIST ac-dc difference working standards. The uncertainties are given to the nearest 0.1 μA/A for consistency, but this precision may not be significant or meaningful

	Reference RSS	Pooled standard deviation	Comparator and sources	Working standard	Bead & RFI	Proximity effect	Repro-ducibility	RSS uncertainty	Expanded uncertainty
1 mA	6.1	3.0	2.3	4.0	4.0	1.2	8.1	12.2	24.5
2.5 mA	6.1	1.5	1.2	1.7	2.9	1.2	4.6	8.6	17.2
5 mA	6.1	1.5	1.2	1.7	2.9	1.2	4.6	8.6	17.2
10 mA	8.5	1.5	1.2	1.7	2.9	1.2	4.6	10.5	21.0
25 mA	10.4	1.5	1.2	1.7	2.9	1.2	4.6	12.1	24.2
50 mA	12.0	1.5	1.2	1.7	2.9	1.2	4.6	13.5	27.0
100 mA	14.1	1.5	1.2	1.7	3.5	1.7	5.8	15.9	31.9
250 mA	16.0	1.5	1.2	1.7	3.5	1.7	5.8	17.6	35.2
500 mA	17.6	1.5	1.2	2.3	3.5	1.7	5.8	19.2	38.4
1 A	19.5	1.5	1.2	2.3	3.5	1.7	6.9	21.3	42.5
2 A	21.7	2.0	2.3	2.3	4.0	2.9	6.9	23.6	47.2
3 A	24.0	2.0	2.3	2.9	4.0	2.9	8.1	26.2	52.3
5 A	26.2	2.0	2.3	2.9	4.0	2.9	8.1	28.2	56.3
10 A	29.7	3.0	2.3	4.0	4.6	4.0	11.5	32.9	65.9
20 A	34.1	3.0	2.3	4.0	4.6	4.0	14.4	37.9	75.7

**Table 4:** Uncertainty analysis (in μA/A) for customer current shunt calibrations. The calculations are shown to the nearest 0.1 μA/A for consistency, but rounded to the nearest μA/A for the final expanded uncertainty

	Working standard	Pooled standard deviation	Comparator and sources	Definition	Bead & RFI	Proximity effect	Thermal drift	RSS uncertainty	Expanded uncertainty
1 mA	16.1	2.0	4.0	1.7	4.0	2.3	1.2	17.5	35
2.5 mA	13.6	1.5	2.3	1.7	2.9	2.3	1.2	14.5	29
5 mA	13.6	1.5	2.3	1.7	2.9	2.3	1.2	14.5	29
10 mA	18.1	1.5	2.3	1.7	2.9	2.3	1.2	18.8	38
25 mA	21.8	1.5	2.3	1.7	2.9	2.3	1.2	22.3	45
50 mA	24.9	1.5	2.3	1.7	2.9	2.3	1.2	25.4	51
100 mA	29.2	2.0	4.0	2.3	3.5	3.5	2.3	30.1	60
250 mA	32.8	2.0	4.0	2.3	3.5	3.5	2.3	33.6	67
500 mA	36.0	2.0	4.0	2.3	3.5	3.5	2.3	36.8	74
1 A	39.9	2.0	6.9	3.5	3.5	5.8	3.5	41.4	83
2 A	44.3	3.0	6.9	3.5	4.0	5.8	3.5	45.8	92
3 A	49.2	3.0	11.5	5.8	4.0	11.5	8.7	53.1	106
5 A	53.4	3.0	14.4	5.8	4.0	14.4	8.7	58.3	117
10 A	61.1	3.0	23.1	11.5	4.6	23.1	14.4	71.9	144
20 A	70.1	3.0	26.0	14.4	4.6	26.0	23.1	83.9	168

**Table 5:** Calculated uncertainty and closure of the crossover measurements between the two buildup paths

Applied current	Expanded uncertainty (μA/A)	Closure of crossover (μA/A)
25 mA	18	−2
50 mA	22	−4
100 mA	25	+6
250 mA	28	+2
500 mA	32	−7
1 A	36	−5
2 A	39	0
3 A	43	+5
5 A	48	−1
10 A	53	+9
20 A	57	+24

## Footnotes

1 Certain commercial equipment, instruments, or materials are identified in this paper to foster understanding. Such identification does not imply recommendation or endorsement by the National Institute of Standards and Technology, nor does it imply that the materials or equipment identified are necessarily the best available for the purpose.
